# Software LS-MIDA for efficient mass isotopomer distribution analysis in metabolic modelling

**DOI:** 10.1186/1471-2105-14-218

**Published:** 2013-07-09

**Authors:** Zeeshan Ahmed, Saman Zeeshan, Claudia Huber, Michael Hensel, Dietmar Schomburg, Richard Münch, Wolfgang Eisenreich, Thomas Dandekar

**Affiliations:** 1 Department of Bioinformatics, Biocenter, University of Würzburg, Würzburg, Germany; 2 Department of Microbiology, University of Osnabrück, Osnabrück, Germany; 3 Department of Bioinformatics and Biochemistry, Technical University Braunschweig, Braunschweig, Germany; 4 Institute for Microbiology, Technical University Braunschweig, Braunschweig, Germany; 5 Lehrstuhl für Biochemie, Technische Universität München, München, Germany; 6 EMBL, Structural and Computational Biology Unit, Heidelberg, Germany; 7 Department of Neurobiology and Genetics, Biocenter, University of Würzburg, Würzburg, Germany; 8 Institute of Molecular and Translational Therapeutic Strategies, Hannover Medical School, Hanover, Germany

## Abstract

**Background:**

The knowledge of metabolic pathways and fluxes is important to understand the adaptation of organisms to their biotic and abiotic environment. The specific distribution of stable isotope labelled precursors into metabolic products can be taken as fingerprints of the metabolic events and dynamics through the metabolic networks. An open-source software is required that easily and rapidly calculates from mass spectra of labelled metabolites, derivatives and their fragments global isotope excess and isotopomer distribution.

**Results:**

The open-source software “Least Square Mass Isotopomer Analyzer” (LS-MIDA) is presented that processes experimental mass spectrometry (MS) data on the basis of metabolite information such as the number of atoms in the compound, mass to charge ratio (m/e or m/z) values of the compounds and fragments under study, and the experimental relative MS intensities reflecting the enrichments of isotopomers in ^13^C- or ^15^ N-labelled compounds, in comparison to the natural abundances in the unlabelled molecules. The software uses Brauman’s least square method of linear regression. As a result, global isotope enrichments of the metabolite or fragment under study and the molar abundances of each isotopomer are obtained and displayed.

**Conclusions:**

The new software provides an open-source platform that easily and rapidly converts experimental MS patterns of labelled metabolites into isotopomer enrichments that are the basis for subsequent observation-driven analysis of pathways and fluxes, as well as for model-driven metabolic flux calculations.

## Background

Metabolism is central for all cellular processes including adaptation of organisms to their respective life style and conditions. Triggered by the presence and activity of metabolic enzymes and the metabolite fluxes through pathways, cellular reactions constitute a highly dynamic network that can be rapidly and efficiently modulated in response to environmental changes. A number of theoretical techniques has been established to predict metabolic fluxes [1-4]. Implementing different mathematical parallel and sequential algorithms, several desktop and web based batch and interactive software applications [5] have been also developed towards quantitative metabolic flux analysis and modeling [6].

In contrast, only few methods allow direct determination of metabolic fluxes, one of which is based on *in vivo* experiments using stable isotope labelled precursors, such as ^13^C-glucose or ^13^CO_2_. The transfer of label to the metabolic network and the specific isotope distribution in metabolic products can then be taken as evidence of metabolic pathways and fluxes during the experimental period. However, robust technology is required to quantitatively determine the isotopomer abundances in multiple metabolites. Specifically, experimental intensities of mass signals (typically of silylated derivatives, metabolites and fragments thereof in GC/MS experiments) have to be converted into relative and molar isotopomer abundances.

**Isotopologues** are species of a compound that differ only in their isotopic composition [7]. The term **isotopomer** is a contraction of ‘isotopic isomer‘, grouping isotopologues into those molecules which contain the same number of a specific isotope (e.g. ^13^C) at different positions. As an example, 64 stable carbon isotopologues exist for glucose. Out of these, six species constitute isotopomers with one ^13^C-atom at position 1, 2, 3, 4, 5, or 6. In natural compounds, i.e. obtained from the natural environment, the **natural abundance** is the consequence of the natural isotope abundance (i.e. ca. 1.1% for ^13^C at a given carbon position) that is diverted through the complete population of isotopologues due to statistical reasons. In contrast, increased isotopologue abundances are observed in labelling experiments where isotope-enriched precursors (e.g. ^13^C-labelled) are supplied to the organism under study. This results in the enrichment of specific isotopologues, i.e. on top of the natural abundances, in the metabolic products. The deconvolution of mass intensities yielding **isotopomer enrichment** is the key task of the software described in this manuscript. Notably, mass intensities provide information on the abundances of isotopologues harbouring a specific number of the isotope, i.e. one, two, three, etc. ^13^C-atoms, and therefore, the enrichment of isotopomeric groups (**isotopomer distribution**) is obtained. Since metabolic pathways lead to specific isotopomer enrichments and, as a consequence, to specific isotopomer distributions, the latter values can be used to identify and to quantify the relative contributions of metabolic routes from the labelled precursor to the products observed by MS.

So far, three different methods are available for positional isotopomer determination, nuclear magnetic resonance (NMR), mass spectrometric analysis of a sufficient number of useful metabolite fragments [8,9], and multiple reaction monitoring (MRM). These methods can provide orthogonal information and can be combined using our software to improve positional isotopomer determination [10].

In this manuscript, a new freely available software is described that is capable of providing a user friendly graphical interface for the efficient and independent (no third party application is needed) data storage, management and processing towards mass isotopomer distribution analysis [11]. The implemented software enables the user to load data from previously created data files or add data manually into the software application at run time and to process it. Furthermore, it directly parameterizes input experimental data to Brauman´s algorithm for accurate estimation of natural and relative abundances. No such application exists, implementing similar mathematics into a user friendly software package.

Currently, only commercial software or user-specific approaches are available for the conversion of mass intensities (provided by the specific software implemented to the mass spectrometer) to the relative and molar isotopomer enrichments, such as tandem mass spectrometric data computing for positional isotopomer distributions [12], measurements of mass distributions by mass spectrometry [13], isotopomer analysis using GC-MS [10,14], and GC-MS analysis for isotopomer balancing [15].

However, for a broad range of users, a open-source software compatible to data exchange with the standard mass software packages is highly desirable. Here, we present a new open-source software using Brauman’s least square method for the calculation of isotopomer enrichment that can be used in GC/MS and LC/MS experiments (including tandem-MS/MS) by calculating relative and absolute isotopomer abundances from the mass ratios of signals in experimental MS spectra.

## Implementation

### Algorithm

**Figure 1:**
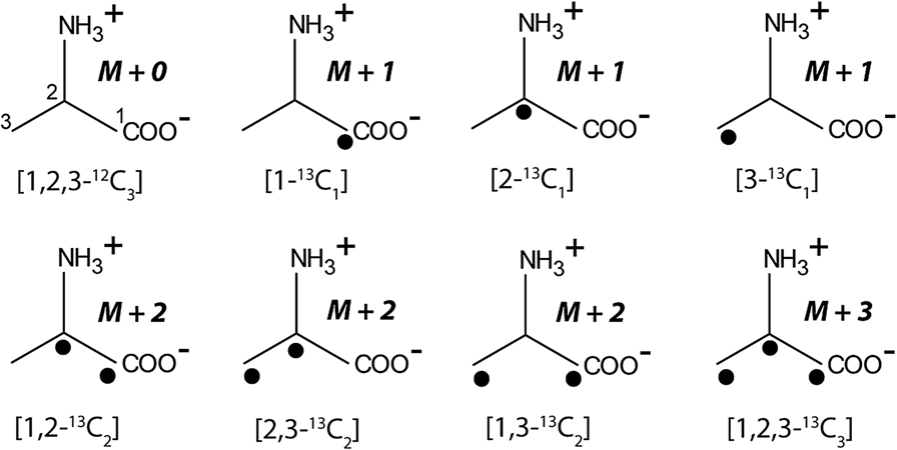
**Stable carbon isotopologues of alanine.** The filled circles indicate 13C-atoms. The mass parameters that are detected by MS are given as M+0, M+1, M+2 or M+3, respectively.

The software treats experimental raw data from MS. Specifically, MS intensities of metabolic products (typically ^13^C- or ^15^ N-labelled) are analysed on the basis of their m/e values and the number of C or N atoms, respectively, in the given molecule, derivative or fragment thereof. Overall ^13^C- or ^15^ N-enrichment and the relative and molar contribution of isotopomers are then calculated using Braumann’s least squares algorithm [16]. Mass distribution measured by MS display enrichments of isotopomer groups (i.e. isotopologues with a given number of the label (Y or 1 in our notation), for example one, two, three etc. ^13^C-atoms). For example, the isotopomer distribution of the C_3_-compound alanine (see Figure 1) is calculated from the abundances of the unlabelled compound (e.g. 000 for three ^12^C-atoms), of the isotopomer group containing one label (00Y, where Y can be at any carbon position), two labels (0YY, the ^12^C-atom 0 can be again at any position in alanine), and three labels 111.

The software is composed of two parts: (i) generation of an appropriate set of linear simultaneous equations and (ii) solution of these equations. The proposed matrix equation to calculate isotopomer enrichments is the least square method using the Moore–Penrose pseudo inverse (the latter is a powerful mathematical method for matrix calculations, nothing specific to isotopomers or metabolism):

(1) $$X={\left(A^{T}*A\right)}^{-1}A^{T}P$$

where X = calculated relative intensities of the fragments, A = matrix of relative natural abundance values for all possible isotopomers and P = set of the experimental relative intensities of the fragments (observed during experimentation). The used mathematics follows Brauman’s approach [16] and recommended considerations for its application [14,17]. The number of C atoms in the fragments contributes to relative natural abundance distributions. It is nevertheless worth while to look for contaminating fragment ions in the mass spectrum, as its impact is substantially corrected through the subtraction of relative natural abundance values. The proposed binomial expression [14] calculating isotopomer fragment distribution taking into account relative natural abundance is

(2) $$A=n!/\left[\left(i!\right)*\left(n-i\right)!\right]*P_{o}{}^{\left(n-i\right)}*P_{1}{}^{i}$$

where A = relative natural abundance, n = number of carbon atoms, i = index variable to count n iterations. P_0_ and P_1_ stand for the abundance of ^12^C and ^13^C, respectively.

“The solution of these equations gives the abundance of each organic moiety. Because of the way in which the problem was formulated, the total abundance of the organic moieties must remain constant.” This statement by Brauman [16] has now to be put into practical calculations. However, the results of this technique depend upon a number of factors: (i) the analysis is based on the assumption that the fragmentation patterns for all heteroatom isotopes are identical (i.e., no differential isotope effect), (ii) the experimental relative abundance of ^12^C and ^13^C isotopes induced through derivatization is known and (iii) the relative natural abundances of the isotopes are either known or measured. The LS-MIDA software package itself is not designed to perform integration of the original MS signals. Thus, another software package must be used first. For our examples, it relied on pre-processing by the software LabSolutions by Shimadzu which is standard software directly supplied with the instrumentation for GC-MS. However, any type of pre-processing software can be used in combination with LS-MIDA.

To predict the relative isotopomer contributions in natural abundance compounds, linear regression analysis is performed by drawing an abundance matrix (eq. 3) using the known or estimated natural abundance values of isotopes by binomial expression.

(3) $$A=\left(\begin{array}{l} \begin{array}{ccc} A_{1} & 0 & \begin{array}{cc} 0 & 0 \end{array} \end{array}\\ \begin{array}{cc} \begin{array}{cc} \begin{array}{cc} A_{2} & A_{1} \end{array} & 0 \end{array} & 0 \end{array}\\ \begin{array}{cc} \begin{array}{cc} \begin{array}{cc} A_{3} & A_{2} \end{array} & A_{1} \end{array} & 0 \end{array}\\ \begin{array}{cc} \begin{array}{cc} \begin{array}{cc} A_{4} & A_{3} \end{array} & A_{2} \end{array} & A_{1} \end{array}\\ \begin{array}{cc} \begin{array}{cc} \begin{array}{cc} \ldots & \ldots \end{array} & \ldots \end{array} & \ldots \end{array}\\ \begin{array}{cc} \begin{array}{cc} \begin{array}{cc} A_{n} & A_{n-1} \end{array} & A_{n-2} \end{array} & A_{n-3} \end{array} \end{array}\right)$$

Here, A_1,_ A_2_, A_3,_ A_4_ … A_n_ are the estimated relative natural abundance values of fragments using eq. 2. These values are used in the abundance matrix A [17,18], based for linear regression analysis. In general, obtaining the coefficients of matrix A (eq. 3) is the non-trivial part of the method. More specifically, the atoms and their isotope distributions from the derivatisation agent must be taken into account, if present. Overlaps of mass traces by impurities have obvious effects and the mode of ionization (positive or negative) has effects on the m/z values. Our implementation tackles the processing of the pre-processed data to overcome some of these problems. For example, contributions due to the derivatisation agent are filtered out.

Next, Brauman’s least square algorithm (eq. 4) is applied for the estimation of relative intensity values for the fragment spectrum:

(4) $$Ri_{\left(1-n\right)}=A^{-1}*P$$

Here, Ri_(1-n)_ are the string of predicted relative intensity values with respect to the m/e values. The length of the abundance matrix depends upon the total number of m/e measurements and experimental relative intensity values. The set of linear equations used to draw the abundance matrix and multiplications for quantitative analysis [14] is given in eq. 5.

(5) $$\begin{array}{l} S_{0}T_{0}\;=U_{0}\\ S_{1}T_{0}+S_{0}T_{1}+\;=U_{1}\\ S_{2}T_{0}+S_{1}T_{1}+S_{0}T_{2}\;=U_{2}\\ \ldots \ldots \ldots \\ \ldots \ldots \ldots \\ S_{n}T_{m‒1}+S_{n‒1}T_{m}\;=U{\;}_{\left(n+m‒1\right)}\\ S_{n}T_{m}\;=U{\;}_{\left(n+m\right)} \end{array}$$

Here, the linear regression analysis (initially used by Brauman [16]) is performed for spectral data analysis, where U is the mass isotopomer distribution, and S and T are the isotope abundances for ^12^C and ^13^C, respectively.

To compute isotopomer abundances for each fragment, again linear regression analysis is performed by calculating the abundance matrix, but the input values are now the observed relative intensity values (**Ri**_**(1-n)**_) in the MS-traces. The length of the abundance matrix depends on the number of fragments with the result:

(6) $$\mathrm{Abundance}\;\mathrm{Matrix}\;\left(Ri_{\left(1‒n\right)}\right)=\left(\begin{array}{l} \begin{array}{cc} \begin{array}{cc} \begin{array}{cc} Ri_{1} & 0 \end{array} & 0 \end{array} & 0 \end{array}\\ \begin{array}{cc} \begin{array}{cc} \begin{array}{cc} Ri_{2} & Ri_{1} \end{array} & 0 \end{array} & 0 \end{array}\\ \begin{array}{cc} \begin{array}{cc} \begin{array}{cc} Ri_{3} & Ri_{2} \end{array} & Ri_{1} \end{array} & 0 \end{array}\\ \begin{array}{cc} \begin{array}{cc} \begin{array}{cc} Ri_{4} & Ri_{3} \end{array} & Ri_{2} \end{array} & Ri_{1} \end{array}\\ \begin{array}{cc} \begin{array}{cc} \begin{array}{cc} \ldots & \ldots \end{array} & \ldots \end{array} & \ldots \end{array}\\ \begin{array}{cc} \begin{array}{cc} \begin{array}{cc} Ri_{n} & Ri_{n‒1} \end{array} & Ri_{n‒2} \end{array} & Ri_{n‒3} \end{array} \end{array}\right)$$

In eq. 6, Ri_1_ Ri_2_ Ri_3,_ Ri_4 ,…_ Ri_n_ are the observed relative intensity values with respect to the m/e values. Now, eq. 1 is applied to estimate the string of calculated relative abundance values (Ra_(1-n)_) for the fragments, where A = Abundance Matrix (**Ri**_**(1-n)**_).

With eq. 7 absolute ^13^C enrichments are then calculated:

(7) $$\mathrm{Ab}s^{13}C=\left(\sum A_{0\ldots n}*n\right)/a$$

The absolute ^13^C-enrichment is equal to the sum of all labeled isotopomers multiplied with the respective number of labels (0 to n), divided by the number of carbon atoms in the fragment under study. A indicates the labeled isotopomer, the index 0 till n indicates the number of labeled atoms in the fragment, and a indicates the number of carbon atoms in the fragment. The matrix calculations are mathematical simple, however, the optimized combination of pre-filtering software and abundance calculations by LS-MIDA takes into account all required steps as well as experimental complications (e.g. filtering out derivatization agent) in a single user-friendly and open-source software package.

## Development

The above described mathematics and calculations were implemented into the software “LS-MIDA” (executable available as Additional file 1; test data in Additional file 2; pre-filtering software considerations see above). We show that the implementation of Brauman’s least square method and the inclusion of binomial expression allow accurate calculations of isotopomer enrichments using experimental GC/MS data of ^13^C-labelled silylated amino acids. LS-MIDA is a UML designed [19] and successfully evaluated third party tool independent reusable desktop application (batch) with user friendly graphical interface, capable of sequentially processing standard input and producing visual output presentation (text and spectrum).

**Figure 2:**
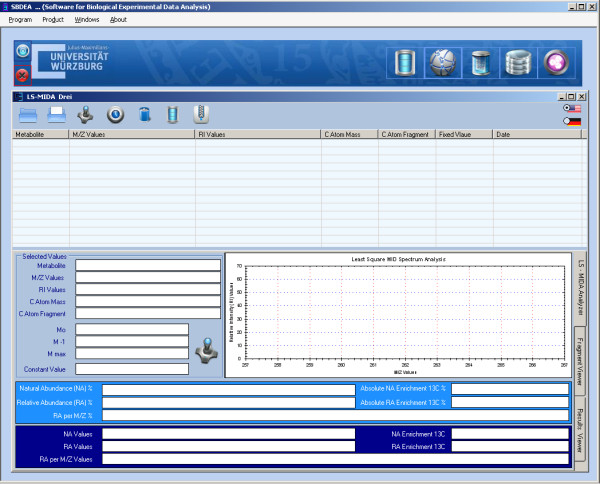
**LS-MIDA for isotopomer distribution analysis.** The Data Analyzer to be loaded with the experimental data; manual entered data and/or created data files using the Data Manager.

**Figure 3:**
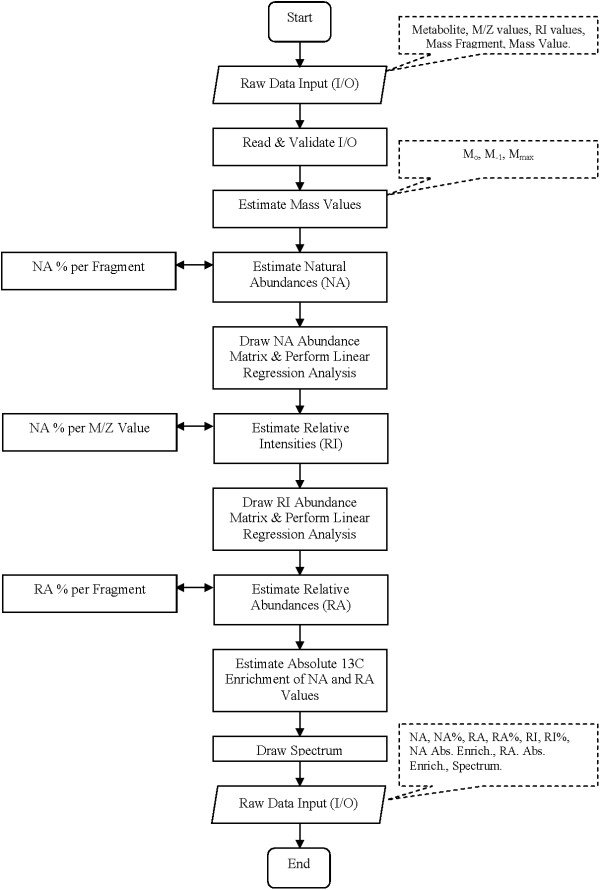
**LS-MIDA; Flow Chart.** Visual presentation of the unified mark-up language (UML) based flow chart. The implemented flow of operations performed during experimental data input, processing, analysis and visualization is given.

The available and tested version of LS-MIDA provides two main modules, the data analyzer (see Figure 2) and the data manager (see Additional file 3, installation and technical overview). The data analyzer is capable of processing input data (metabolite information, m/e values and experimental relative MS intensity values). It then estimates mass values (M_***o***_, M_***-1***_, M_***max***_), predicts relative natural abundance values, and calculates the actual isotopomer abundances from the MS patterns. Finally, it allows drawing the isotopomer distribution of the calculated values (see Figure 3).

### Graphical user interface

LS-MIDA provides an intuitive graphical user interface for file based experimental raw data manipulation and management. It is capable of not only managing user input experimental raw data but also provides options to manage resultant data (output of LS-MIDA). It allows the user to create new data files, manage created data files, merge new or already made data files into one or more new or already created data files and manipulate entries of data files. It is an independent file based data management system that does not require any external or third party database to install and use. LS-MIDA is implemented and tested using Microsoft Windows (version XP and 7) operating system as it is developed using Microsoft Dot net framework and C Sharp (object oriented) programming language. The licensed software is freely available for academic use on request.

### Database manager

LS-MIDA advantageously provides a file-based data management system for experimental metabolic mass isotopomers based raw data. The data manager is a supporting utility, developed as a user-friendly file-based experimental data management system. It allows the user to create new experimental data files that later can be used for the analysis using data analyzer. The experimental data is organised following a new data format especially proposed (with extension “*.ls”) for LS-MIDA data files. Data manager allows the user to read, add, edit, update, delete and merge data (from other source files of the same extension) into a file.

## Results

### Calculations

**Figure 4:**
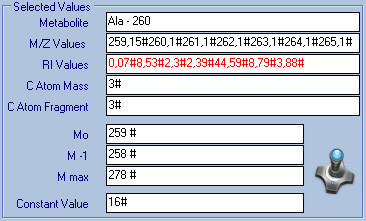
**LS-MIDA; Input Interface.** This figure presents input information (example: alanine; Metabolite name, m/e values, R_i_ values, C Atom Values, C Atom fragment values) and estimated mass values (M_0_, M_1_, M_max_) for Ala 260. The input and resultant values are also presented in Table 1.

The implemented mathematical procedure in LS-MIDA version 3.0 (see Figure 4) starts with the input (I/O) of metabolite information (e.g., name) and of the raw data from MS spectra, i.e., m/e values, and the experimental relative intensity (R_i_) values. After I/O validation, the mass values M_*o*_, M_*-1*_, M_*max*_ are calculated to adjust the potential mass distribution.

**Figure 5:**
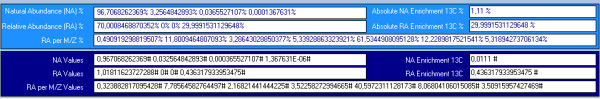
**LS-MIDA; Calculation.** Estimated natural abundance, relative abundance, relative intensity values and absolute enrichments for alanine, fragment weight is 260.

Then, using binomial expansion, relative natural abundances N_a_ with percentages per each fragment are estimated (see Figure 5). Next, linear regression analysis is performed and the abundance matrix is drawn with the application of Brauman’s least square method using estimated N_a_ values to derive the relative intensity values per m/e value for natural abundance compounds. The relative intensity values R_i_ are then used to calculate the isotopomer abundances R_a_ (including their percentage amounts in each fragment) for the labelled compounds under study.

**Figure 6:**
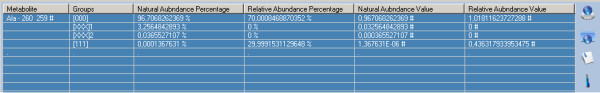
**LS-MIDA; Abundance.** Calculated percentages of abundance values for Alanine in fragment groups.

For this purpose, once again a linear regression analysis is performed drawing the abundance matrix with the implementation of Brauman’s least square method. Using the calculated N_a_ and R_a_ values, absolute ^13^C enrichment is then calculated for each fragment (see Figure 6).

**Figure 7:**
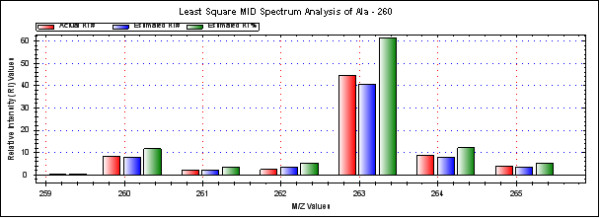
**LS-MIDA; Spectrum.** Alanine Mass Spectrum (R_i_ to m/e). Spectrum is drawn and shows estimated relative intensity values, based on the input experimental intensity values with respect to the m/e values. All data can be processed at once (click the button “Measure all the data”).

The output (N_a_ and R_a_ values) is presented in numeric format and in special notation format (based on the number of C atoms in the fragments) and the isotopomer distribution is shown graphically (see Figure 7).

### Application in metabolite measurements

The software was now tested with different data sets. ^13^C-Labelled amino acid samples (analyzed as TBDMS-derivatives) were obtained from hydrolysates of *Salmonella enterica* grown in medium containing [U-^13^C_6_]glucose [20]. We have shown earlier that [U-^13^C_6_]glucose is efficiently incorporated into most amino acids of *Salmonella enterica* via intermediates of glycolysis. Under these conditions, alanine is mainly composed of the unlabelled isotopomer (derived from unlabelled glucose in the medium) and the fully ^13^C-labelled isotopomer due to *de novo* synthesis of alanine from [U-^13^C_3_]pyruvate made from [U-^13^C_6_]glucose via glycolysis.

As an example for the involved numerical steps, Table 1 and Figure 4 show input parameters and experimental MS raw data of three fragments for labelled TBDMS-alanine such as metabolite information, m/e values of the relevant fragment, experimental intensity values, atomic mass values and the number of atoms in the fragment. During input, data file preparation, and management, the data manager structures data into experimental data files which are then used by data analyzer for the calculations. The obtained results are shown in Table 1b, Table 1c, and Figures 4, 5 and 6. Observed results are M_***o***_, M_***-1***_, M_***max***_ (the range of mass in which to pick the correct intensities), the predicted relative natural abundances, and the relative abundances of isotopomers in the labelled sample with its absolute ^13^C enrichment

**Table 1:** Calculation results

**(A) Experimental raw data of alanine (Ala)**								
**Metabolite**	**m/e values**	**R**_**i**_**values**	**C Atom Metabolite**	**C Atom Fragment**				
Alanine (Ala)	259.15#260.1#261.1#262.1#263.1#264.1#265.1#	0.07#8.53#2.3#2.39#44.59#8.79#3.88#	3	3				
**(B) Alanine experimental raw data analysis**^1^								
Metabolite	M_o_	M_-1_	M_max_	N_a_	R_a_	N_a_ Abs.	R_a_ Abs.	R_i_ values
						**Enrichment**	**Enrichment**	
Alanine (Ala)	259	258	278	0.967068262369#	1.01811623727288#	0.0111 #	0.436317933953475 #	0.323882817095428#
				0.032564842893#	0#			7.78564582764497#
				0.000365527107#	0#			2.16821441444225#
				1.367631E-06#	0.436317933953475#			3.52258272994665#
								40.5972311128173#
								8.06804106015085#
								3.50915957427469#
**(C) Isotopomer calculation results (example: alanine)**								
**Isotopomeric group**	**N**_**a**_**%**	**R**_**a**_**%**						
[000]	96.7068262369%	70.0008468870352%						
[XXX] 1	3.2564842893%	0%						
[XXX] 2	0.0365527107%	0%						
[111]	0.0001367631%	29.9991531129648%						

^1^ Here and in the following long (many digits) calculation results are shown to illustrate arithmetics.

As shown in Figure 7, abundances of four different alanine isotopomers are observed. For the isotopomer 000 (i.e. ^12^C only), the estimated natural abundance is 96.7%, whereas the same isotopomer accounts for 70.0% in the labelled compound. The isotopomer group with one ^13^C-atom has 3.26% or 0% abundance in the unlabelled or labelled compound, respectively. The relative abundances for the isotopomer group comprising two ^13^C-atoms are 0.037% or 0% in the unlabelled or labelled alanine sample, respectively, whereas the abundances for the fully labelled isotopomer are 0.00014% or 30.0% in the unlabelled or labelled sample, respectively. On this basis, the observed absolute enrichment value of ^13^C in the labelled sample from *S. enterica* results in 30.0%. This is in line with our expectations and calculations also using other software tools for isotopomer analysis.

The resulting spectrum is shown in Figure 7. At constant m/e values the peaks of the drawn spectrum may vary according to their molecular composition [16]. The strongest observed relative intensity ^13^C isotopomer peak in this example is at 263.1 in the correct range of M_*o*_, M_*-1*_, M_*max*_ so the values for 260.1, 263.1 and 264.1 are shown in Figure 7 for the alanine mass spectrum. For more results of labelled TBDMS-amino acids from the same labelling experiment [20,21], please have a look at Additional file 3: Table S2-S3.

For the pathway analyses of Salmonellae mentioned above and in similar studies on other bacteria, we tested the processing of different data sets; input data file preparation and management, experimentation and data analysis. This allowed also an analysis of error rates. Accuracy of the calculation itself is quite high (error less then 1 part per billion). This estimate is based on several hundred test runs of the software and meticulous testing for bugs and unexpected behavior. Regarding errors from the non-trivial coefficients of matrix A involving experimental errors from atoms of the derivatization agent present and overlap of fragment spectra as well as the mode of ionization (positive or negative), we expect error rates of less than 1%.

Another inherent source of error is the analysis of network fluxes for complex biological system that typically result in notably higher deviations when looking at the results from replicates. As these are indirectly inferred from the changes of measured isotopomer concentrations, these data already carry the error due to the mathematical procedures. In addition, experimental errors regarding growth conditions must be taken into account (cell number, actual glucose concentration, sample preparation). Indeed, the resulting overall error for flux analyses considering all these effects can be as high as 10% based on our experience. Some limitations arise from the fact that not in all pathways metabolized label is present, but this is an inherent prediction limitation for the approach.

## Discussion

^13^C Labelling of metabolites has proven to be a powerful method in quantifying metabolic routes and fluxes, especially, if there are alternative pathways between two metabolites. Isotopomer balancing provides the basis for deducing metabolic pathways and fluxes.

In own studies, we have shown that ^13^C-incorporation studies coupled to software-based isotopomer calculation allowed us to identify the pathways of amino acid biosynthesis [22] under relevant non-standardized conditions (e.g. proline biosynthesis in *Listeria monocytogenes* and its modulation by the transcription factor PrfA [21]). Another example concerns nutrient supply for *Salmonella* in the *Salmonella* containing vacuole [20]. Again, the unequivocal determination of nutrient flow across the vacuole to *Salmonella* depends on isotopologue data with the use of suitable processing software.

In general, without transforming the mathematics into applied software doing the calculations, none of these and other insights [6,20,21] regarding flux modifications and usage of different metabolic pathways in different organisms is possible.

Despite this potential power of the methodological approach, it is still difficult to perform metabolic flux analyses due to the lack of user-friendly and open-source software tools. This limitation also demands the development of mathematical modelling of metabolism for each substrate to obtain more detailed and accurate results. Before the implementation of LS-MIDA, we relied on the usage of a lab-specific Excel/Solver-based software doing the required calculations. However, this approach did not provide user-friendly output formats nor included a database allowing extensive comparative studies. As an alternative to this lab-specific solution, commercial packages may also be used [12,13,15]. However, these software packages are not freely available.

In order to establish tools that can be widely distributed, we have established the LS-MIDA software. Briefly, Brauman´s least square algorithm is used and developed in the form of a versatile software application iteratively analyzing the estimated abundance resonances [23] after binomial expansion for the calculation of isotopomer enrichments in labelled metabolites.

Furthermore, LS-MIDA provides a file-based data management system for fast and accurate MS-based isotopomer analyses.

In comparison with other existing approaches [24-26], the combined features in LS-MIDA are not available in standard packages for metabolite modelling such as Metatool [18], Yanasquare [27], Gepasi [28] or FiatFlux [1] (here fluxes are predicted after the isotopologue data have been processed). There are two software solutions available for isotopomer data processing, Envelop [24] and Isotope Pattern Calculator [25], but none uses binomial expression for data extension. The implementation of Brauman’s least square method with the inclusion of binomial expression allows rapid and accurate calculation of isotopomer data.

LS-MIDA was compared with in-house software demonstrating its robustness. This showed that LS-MIDA can be used as an open-source platform for many (even non-expert) users in consortia in research programs such as the ongoing priority DFG program dealing with “host adapted metabolism of pathogens” in our example as well as other interested academic groups (German and foreign) for which we did provide and also will provide in future the software free of charge. Training courses and service are offered to support the use of the software within the program and for other users. In consequence, the software is freely available for the world-wide academic community.

## Conclusions

LS-MIDA is a versatile, open-source, and user friendly software with rapid calculation, integrated overview on all isotopomers, least square correction and database management system, with good application potential for biology and biotechnology such as studying the core metabolism of organisms, the pathways and fluxes leading to desired products in biotechnology, and complementing methods from genomics, proteomics or metabolomics.

## Availability and requirements

LS-MIDA is free available software for all academic users with open license; a commercial license can be obtained on request.

LS-MIDA is developed using the Microsoft C# (sharp) programming language and Microsoft Dot Net framework 2008. It is compatible (install and use) for all Microsoft Windows operating systems. Moreover, LS-MIDA automatically adopts the language of the installed operating system and presents numerical values accordingly e.g. in case of English language decimal values are ‘.’ (dot) separated and in case of German language decimal values are ‘,’ (comma) separated. Numerical values are separated by ‘#’ (hash) symbol for all languages.

Further details are available in the Additional file 3 Tutorial (installation, evaluation, further data, glossary).

## Competing interests

The authors declare no competing interest (no reimbursements, fees, funding, or salary from an organization that may in any way gain or lose financially from the publication of this manuscript, either now or in the future, stocks or non-financial competing interests (political, personal, religious, ideological, academic, intellectual, commercial or any other).

## Authors’ contributions

ZA: software designing, programming and testing. SZ assisted ZA. DS, RM, TD provided software expertise. CH, WE, MH provided experimental expertise. TD and WE lead and guided the study. All authors (ZA, SZ, CH, MH, DS, RM, WE, TD) participated in data analysis and evaluation of the software as well as in writing of the manuscript. All authors read and approved the final manuscript.

## Supplementary Material

Additional file 1 **LS-MIDA software application.** An executable software file (setup) is included. Click here for file

Additional file 2 **Example data for different amino acids.** Example data for different amino acids analyzed by LS-MIDA. Click here for file

Additional file 3 Tutorial, installation, evaluation, further data, glossary. Click here for file
