# Supplementary material for: Software LS-MIDA for efficient mass isotopomer distribution analysis in metabolic modelling
Source: BMC Bioinformatics. 2013 Jul 9;14:218. doi: 10.1186/1471-2105-14-218 (PMC3720290; doi:10.1186/1471-2105-14-218)
Supplement: Additional file 3 — Tutorial, installation, evaluation, further data, glossary. [file 1471-2105-14-218-S3.doc]

# Supplementary Material:

# Software LS-MIDA for efficient Mass Isotopomer Distribution Analysis in Metabolic Modelling

### Zeeshan Ahmed^*, 1,7,†^, Saman Zeeshan^1, 8,†^, Claudia Huber^5^, Michael Hensel^2^, Dietmar Schomburg^3^, Richard Münch^4^, Wolfgang Eisenreich^5^ and Thomas Dandekar^§, 1,6^

^1^Department of Bioinformatics, Biocenter, University of Würzburg, Germany, ^2^Department of Microbiology, University of Osnabrück, Germany,

^3^Department of Bioinformatics and Biochemistry, Technical University Braunschweig, Germany,

^4^Institute for Microbiology, Technical University Braunschweig, Germany,

^5^Lehrstuhl für Biochemie, Technische Universität München, Germany,

^6^EMBL, Bioinformatics Heidelberg, Germany,

^7^Department of Neurobiology and Genetics, Biocenter, University of Würzburg Germany,

^8^Institute of Molecular and Translational Therapeutic Strategies, Hannover Medical School, Germany

^†^ Equal contributors.

^§^Corresponding author

Email addresses:

ZA: [zeeshan.ahmed@uni-wuerzburg.de](mailto:zeeshan.ahmed@uni-wuerzburg.de)

SZ: [saman.majeed@uni-wuerzburg.de](mailto:saman.majeed@uni-wuerzburg.de)

CH: [claudia.huber@mytum.de](mailto:claudia.huber@mytum.de)

MH: [michael.hensel@biologie.uni-osnabrueck.de](mailto:michael.hensel@biologie.uni-osnabrueck.de)

DS: [d.schomburg@tu-bs.de](mailto:d.schomburg@tu-bs.de)

RM: [r.muench@tu-bs.de](mailto:r.muench@tu-bs.de)

WE: [wolfgang.eisenreich@mytum.de](mailto:wolfgang.eisenreich@mytum.de)

TD: [dandekar@biozentrum.uni-wuerzburg.de](mailto:dandekar@biozentrum.uni-wuerzburg.de)

# Installation

- Take the setup executable SBEDA (Software for Biological Experimental Data Analysis) framework.

- To run LS-MIDA Data Analyzer click on the “Blue Globe” icon in the main bar and to run Data Manager click on “Cylinder Database” icon.

- For a simple example and guided tour, load the example data into Data Analyser by clicking the “Open Data File” icon, and process these by clicking “Measure Selected Data” icon.

- To obtain a spectrum click on the white control. Complete software details are provided in Table 3.

- The SBEDA framework (including LS-MIDA) is developed using the Microsoft C# (sharp) programming language and Microsoft Dot Net framework 2008.

It is compatible (install and use) for all Microsoft Windows operating systems. The SBEDA framework automatically adopts the language of the installed operating system and presents numerical values accordingly e.g. in case of English language decimal values are ‘.’ (dot) separated and in case of German language decimal values are ‘,’ (comma) separated. Numerical values are separated by ‘#’ (hash) symbol for all languages.

# Short Tutorial

**User Interface:** The graphical user interface of LS-MIDA Data Analyzer consists of ten controls: open data file, clear all text controls, measure selected data, measure all data, delete selected data, open data manager, exit, languages, present input data and selected values.

Furthermore, the graphical interface is divided into three views: LS-MIDA Analyzer (Figure 1a), Fragment Viewer (Figure 1b) and Result Viewer (Figure 1c). The data shown are also listed in detail in Table 3. These describe accurately and step by step every option available for the user and how to process and analyse the data. More information is available on request from the authors. The software distribution comes with a data-example regarding amino acid production in Salmonella strains. To process the data, load down the example data (example.dat). The graphical user interface of LS-MIDA Data Manager consists of 15 controls: open data file, clear all text controls, close isotopo data manager, add new values, update edited values, clear text fields, save data in file, select values to edit, delete values, create new data file, select source directory, save file, cancel creating file, data view and language as shown in Figure 2 and described in Table 4.

# Technical Overview

LS-MIDA is a multiple document interface (MDI) software application (public and freely distributed for academic use), developed following the principle of embedding children windows under a single parent window by creating nested hierarchies. To meet aforementioned goals of LS-MIDA development, the graphical user interface of this application is divided into two main modules i.e. Data Analyzer and Data Manager. Data analyzer is responsible for providing options for experimental data load, analysis and visualization, whereas data manager is for biological experimental data manipulation and management.

The graphical user interface of data analyzer consists of eight main features i.e. language, control box, data view, selected values, analysis, calculated abundances, calculation log and visualization, as shown in Figure. 1 (a, b, c). These options help in loading experimental data, analyzing it and visualizing results. The graphical user interface of data manager consists of seven main options i.e. language, file control box, file information, create new data file, experimental data, selected data and data manipulation options, as shown in Figure 2. These options allows user to create new data files, manage created data files, merge new or already made data files into one or more new or already created files data files and manipulate entries data files. It is an independent file based data management system, it does not require any external or third party database to install and use.

Different mathematical algorithms (binomial expansion, Brauman’s least square, absolute 13C Enrichment, quantitative linear regression and abundance matrix) are used to measure mass isotopomers distribution from spectral data by analyzing each peak of given mass and each mass atom fragment. These algorithms are capable of analyzing (isotopic) labelled (carbon) metabolite based experimental raw data (different amino acids) by measuring mass values (minimum and maximum). They predict natural abundance values for pure compounds, calculate and draw linear relationships between masses within the range of a compound and perform linear regression analysis to predict relative intensity values R_i_ with respect to each m/z value.

Binomial expansion uses actual (observed during GC-MS experimentation) r_i_ values and number of mass fragments to measure natural abundance values. Brauman’s least square method is used to compute R_i_ values per each m/z values. Computed natural abundance values and R_i_ values are used in quantitative linear regression analysis to predict relative abundance values and their percentages per each mass fragment. The absolute ^13^C enrichment method is used to calculate absolute natural abundance enrichment and absolute relative abundance enrichments.

We compared different experimental raw data on amino acids with their m/z values, actual R_i_ values per each m/z value, number of C atoms in respective fragments, number of C atoms in compound and mass values.

# Processing Experimental Data

To apply LS-MIDA, experimental data have to be collected first. Data processing using LS-MIDA consists of three steps: preparation of data set, input data file preparation and management, and data analysis. Observed data during actual experimentation (GC-MS) are collected during the preparation of data set. During input data file preparation and management at first data manager is used to structure data by organizing in an experimental data file which is later used by data analyzer for analysis. Throughout the experimental data analysis, each amino acid is individually analyzed.

Observed results after different experiments of metabolic isotopomers analysis with different metabolites using GC-MS are collected for mass isotopomers predictions using LS-MIDA. The collected data consists of five different amino acids i.e. Glycine (Gly – 246), Lysine (Lys- 300), Aspartic Acid (Asp- 418), Threonine- 404, Proline (Pro-184), described in Table 1; consists of following experimental elements i.e. m/z values, RI values, atomic mass values and atomic fragment numbers. A data input file is created using data manager, as shown in Figure 2. The sequence of input file creation started with the creation of a new file and entering manually each metabolite based experimental data. Before starting analysis using data analyzer, newly created input data file is validated by data analyzer and as shown in Figure. 3 (a, b, c).

During experimental data analysis, the prepared data file is input and analyzed using data analyzer item by item. Each input item of experimental data consists of five main information elements i.e. metabolite name, m/z values, R_i_ values, atomic mass values and atomic fragment numbers. Evaluation process using data analyzer starts with the use of data entries of earlier mentioned complete experimental data set. The data entry of each metabolite (Gly, Lys, Asp, Thr, Pro) is selected and processed for analysis (one by one or at once all) as shown in Figure 3a. The resultant information from data analyzer consists of three different items i.e. natural abundances, relative abundances (Figure 3b) and percentage of calculated relative abundances per m/z values, in each experiment (Figure 3c), described in Table 2. Furthermore based on observed results spectra are drawn as shown in Figure 4a-e.

# Evaluation

The methodology (see above) includes statistical calculations and we give the sequence of steps for the implementation process. We tested different data sets; input data file preparation and management, experimentation and data analysis. Example data and results presented here are from Salmonella labelling experiments for different metabolites including analysis of various amino acids or central carbohydrate metabolites. During input data file preparation and management the data manager structures data into experimental data files which are later used by data analyzer for analysis. Throughout the experimental data analysis, each observed resultant data set during experimentation is individually analyzed using LS-MIDA. Data analyzer and individual results are presented in Table 1S and Table 2S. From two software solutions available for isotopologue data processing i.e. Envelop [1] and Isotope Pattern Calculator [2], none uses binomial expression for data extension.

The implementation of Brauman’s least square method with the inclusion of binomial expression allows the isotopologue data to be more accurate. We provide a file based data management system for experimental metabolic mass isotopomers based data. There is fast processing speed (only seconds), calculation complexity scales quadratically with O(n^2^) depending on the number of carbon atoms per isotopologue. ^15^N isotopologue data can also be processed using LS-MIDA.

All data examples given are from Salmonella isotopologue measurements. Accumulating such measurements and taking further data on their metabolism into account allows insights into Salmonella and their metabolism during infection (Figure 5; reference [3]). The diagram (Figure 5) shows flux adaptation in Salmonella and we indicate from which part of the metabolic flux map the examples given are derived. For instance, the data on alanine measurements provide information on this branch of the map and can be processed following the short tutorial.

Glucose, glucose-6P and gluconate present possible carbon sources for intracellular pathogens. For such carbon sources the enzymes and fluxes for glycolysis and for the Entner–Doudoroff pathway are up-regulated in these bacteria. In contrast, most enzymes and the fluxes in the TCA cycle are down-regulated.

The Salmonella model derived after processing the data (Figure 5) and complementing this by further experiments and information shows furthermore that in Salmonella preferred carbohydrates are glucose and glycerol-3-phosphate. Furthermore, entry points for key amino acids into the metabolism during infection are given (blue boxes). The strength of the fluxes corresponds to the situation when these key carbon sources are there. Investigations in the presence of different amino acids with isotoplogue labeling and their metabolism lead to the data examples given here (Table 1 – see above for detailed processing).

The amino acids given are efficiently metabolized and enter the pathways according to their position on the pathway map.

Currently we investigate the effect of different enzyme mutations on the metabolism, for instance regarding PEP carboxylase [5]. If this enzyme is impaired, no anaplerotic reaction is possible. Similarly, mutations in the lower part of glycolysis are interesting, for instance around the metabolism of serine, alanine and glycerine aldehyde 3 phosphate in Salmonellae [5]. Some of these pathway mutations have already been shown to be important for survival in macrophages in other organisms, such as Listeria [4].

# Glossary

Isotopomers are species having identical elemental compositions but are constitutionally isomeric because of isotopic substitution, also called isotopic isomers or isotopologues.

Abundance is the simplification of mass spectra of molecules containing multiple isotopes in terms of one isotope.

Natural abundance is the complete population of isotopomers in the molecules of a compound (with label derived isotopomers).

Relative abundance is the population of artificially labeled isotopomers (e.g. 13C) in the molecules of a compound. Furthermore the calculated relative abundance simplifies the mass spectra of molecules containing elements with many isotopes

Heteroatom is an atom occurring with more than one abundant isotope

M***_o_***, M***_-1_***, M***_maximum_*** these are the ranges (empirical) where the correct measured intensities from Mass spectrometry should be situated.

# References

1. Michael T Sykes and James R Williamson. **Envelope: interactive software for modeling and fitting complex isotope distributions.** BMC Bioinformatics. **9**, 446, 2008.

2. Massila K., Soong H C., Azlianor A A., Muhammad S S. **Reinforcing the concept of calculating isotope pattern using theoretical isotope generator (TIG)**. WSEAS Transactions on Information Science and Applications, **5**. 949, 2008.

3. Eisenreich, W., Dandekar, T., Heesemann, J.,Goebel, W. **Carbon metabolism of intracellular bacterial pathogens and possible links to virulence**. Nat Rev Microbiol 8, 401-412, 2010.

4. Schauer K, Geginat G, Liang C, Goebel W, Dandekar T, Fuchs TM. **Deciphering the intracellular metabolism of Listeria monocytogenes by mutant screening and modelling.** BMC Genomics. 2010; 11:573.

5. Dandekar T, Astrid F, Jasmin P, Hensel M. **Salmonella enterica: a surprisingly well-adapted intracellular lifestyle**. Front Microbiol. **3**,164, 2012.

Table 1: Experimental Data Set

| No. | Metabolite | m/e values | RI values | C Atom Mass | Fragment |
| --- | --- | --- | --- | --- | --- |
| 1 | Gly - 246 | 245.15#246.1#247.1#248.1#249.05#250.1# | 0.27#61.52#13.38#11.9#1.91#0.72# | 2# | 2# |
| 2 | Lys- 300 | 298.25#299.2#300.2#301.2#302.15#303.15#304.15#305.15#306.15# | 0.79#26.4#100#27.88#13.3#4.59#1.56#0.53#0.16# | 6# | 5# |
| 3 | Asp- 418 | 417.25#418.15#419.15#420.15#421.15#422.15#423.1#424.1# | 0.24#70.49#25.14#13.5#52.81#18#7.44#1.66# | 4# | 4# |
| 4 | Thr- 404 | 404.15#405.2#406.15#407.2#408.2#409.2#410.2# | 60.36#24.02#12.85#95.21#31.94#14.67#3.21# | 4# | 4# |
| 5 | Pro - 184 | 183.25#184.15#185.15#186.15#187.15#188.15#189.1#190.1#191.1#192.1# | 0.28#100#16.73#4.76#0.53#0.09#0.65#0.14#0.07#0.01# | 5# | 4# |

## ^1^individual values are separated by a hash ‘#’ .

## Table 2: Calculated Abundances of input metabolites using LS-MIDA Data Analyzer

| Metabolite/Abundances | Gly - 246 | Lys- 300 | Asp- 418 | Thr- 404 | Pro - 184 |
| --- | --- | --- | --- | --- | --- |
| Natural Abundances | 97.792321% 2.195358% 0.012321% | 94.5718499425015% 5.30765261584471% 0.119152480606485% 0.00133743809761551% 7.506099142245E-06% 1.6850581551E-08% | 95.6333804656704% 4.29378308491836% 0.07229395122246% 0.00054098011836% 1.51807041E-06% | 95.6333804656704% 4.29378308491836% 0.07229395122246% 0.00054098011836% 1.51807041E-06% | 95.6333804656704% 4.29378308491836% 0.07229395122246% 0.00054098011836% 1.51807041E-06% |
| Relative Abundances | 99.8912281171436% 0% 0.1087718828564% | 99.3026844773212% 0.65805237403172% 0% 0.0361341584972893% 0% 0.0031289901498035% | 98.2898154891946% 0% 0% 0.884179441492093% 0.826005069313319% | 95.4306486391055% 0% 0% 0.650776321011312% 3.91857503988317% | 99.9712278159443% 0% 0.0287721840556888% 0% 0% |
| Percentage of Relative Abundances per M/Z Values | 1.80213609628263% 67.4093295593138% 14.8808366101696% 13.0223740883095% 2.10025005063644% 0.785073595288046% | 1.29493119012556% 17.3056208343025% 54.8537432146748% 15.4613301062492% 7.32213400130023% 2.5262267584692% 0.858566818538945% 0.29104752220747% 0.0863995541321148% | 1.73052015748485% 36.2024002594465% 13.0326729659361% 8.0283604597961% 27.1022025851006% 9.26691023856856% 3.79794667763311% 0.838986656034203% | 24.5258042348218% 9.84608905503391% 6.84461974156777% 38.5776284857007% 13.0119545375927% 5.91270814602815% 1.28119579925491% | 3.71315835064298% 78.226340378559% 13.1554719031474% 3.71421169606794% 0.415013297114186% 0.0926173048923881% 0.50958369233405% 0.111142505509892% 0.0546968368314347% 0.00776403490068552% |

## Table 3: Graphical User Interface for LS-MIDA Data Analyzer and its controls

| No. | Features | Descriptions |
| --- | --- | --- |
| 1 | Open data file | Opens directory browser to select input data file from attached repositories and loads data from data file into data viewer. |
| 2 | Clear all text controls | Deletes all loaded data and clears all text controls. |
| 3 | Measure selected data | Process selected data entry (only one at a time) from data view and perform MIDA. |
| 4 | Measure all data | Processes all loaded data (all data entries) at once. |
| 5 | Delete selected data | Deletes selected data entry from data view. |
| 6 | Open data manager | Open LS-MIDA Data Manager’s graphical user interface. |
| 7 | Exit | Closes the LS-MIDA Data Analyzer. |
| 8 | Present input data | Provides textual visualization of loaded data (option1). Allow user to select one or multiple values (option 3, 4 and 5) from loaded data. |
| 9 | Selected Values | Provides text boxes for experimental data manipulation by editing selected input data entry values from data viewer or by entering new experimental data for measurement analysis. It provides following text boxes   1. Metabolite; name of the metabolite. 2. M/Z Values; mass to charge ratio values. 3. RI Values; relative intensity values. 4. C Atom Mass: atom number. 5. C Atom Fragment; number of fragments 6. M0; mass value 7. M-1; mass value minus 1 8. Mmax; maximum mass values 9. Constant Value; set mass value. |
| 10 | LS-MIDA Analyzer | Provides textual visualization of resultant information obtained after data processing, consisting of following elements:   1. Natural Abundance (NA) %; measured percentage of natural abundance with respect to the input number of fragments. 2. Absolute NA Enrichment 13C %; measured percentage of absolute natural abundance 13C. 3. Relative Abundance (RA) %; measured percentage of relative abundance with respect to the input number of fragments. 4. Absolute RA Enrichment 13C %; measured percentage of absolute relative abundance 13C. 5. RA per M/Z%, NA Values; measured percentage of relative intensity values per M/Z values 6. NA Values; measured actual natural abundance values. 7. NA Enrichment 13C; measured actual NA Enrichment 13 C value. 8. RA Values; measured actual relative abundance values. 9. RA Enrichment 13C; measured actual RA Enrichment 13 C value. 10. RA per M/Z Values; actual relative intensity values.   Furthermore, LS-MDIA Analyzer viewer provides visual presentation of measured relative intensity values with respect to the mass to charge ratio values, in a spectrum. |
| 11 | Fragment Viewer | Provides the information about measured abundances (natural and relative) with respect to the number of fragments. It consists of four controls as well   1. Export File; allows user to export measure fragment based output in a new file. 2. Import File; allows user to import already estimated data. 3. Clear Text; allows user to clear the view by deleting all the data. 4. Delete Selected Data; allows user to delete particular (selected) data. |
| 12 | Result Viewer | Provides the information of complete output including Metabolite name, input values (M/Z values, Fragment Number) and measured values (NA, RA and RA/MZ).It consists of four controls as well   1. Export File; allows user to export measure fragment based output in a new file. 2. Import File; allows user to import already estimated data. 3. Clear Text; allows user to clear the view by deleting all the data. 4. Delete Selected Data; allows user to delete particular (selected) data. |

## Table 4: Graphical User Interface LS-MIDA Data Manager and its controls

| No. | Features | Descriptions |
| --- | --- | --- |
| 1 | Open data file | Opens directory browser to select input file from attached repositories and loads data from data file into data viewer. |
| 2 | Clear all text controls | Deletes all loaded in data and clears all text controls. |
| 3 | Close isotopo data manager | Closes the LS-MIDA Data Manager. |
| 4 | Add new values | Add newly entered values in text boxes to data view. |
| 5 | Update edited values | Updates edited values in to data view |
| 6 | Clear text fields | Deletes data a from text controls. |
| 7 | Save data in file | Saves data into file. |
| 8 | Select values to Edit | Allows user to select one value from data view to edit existing values. |
| 9 | Delete values | Deletes selected data entry from data view. |
| 10 | Create new data file | Allows user to create new data (input) file. |
| 11 | Select source directory | Opens directory browser to select the directory to store newly created data file. This option is only enabled and visible when user will click option 10. |
| 12 | Save file | Allows user to save newly created file. It also allows user to save exiting file data e.g. if some data file is also open and user want to creates a new file, then system will ask if he want to merge existing data (in data view) to newly created file or not. This option is only enabled and visible when user will click option 10. |
| 13 | Cancel creating file | Allows user to cancel new file creation process. This option is only enabled and visible when user will click option 10. |
| 14 | Data view | Provides the textual view of all loaded, added or updated data. |

# Figures

**(A)**


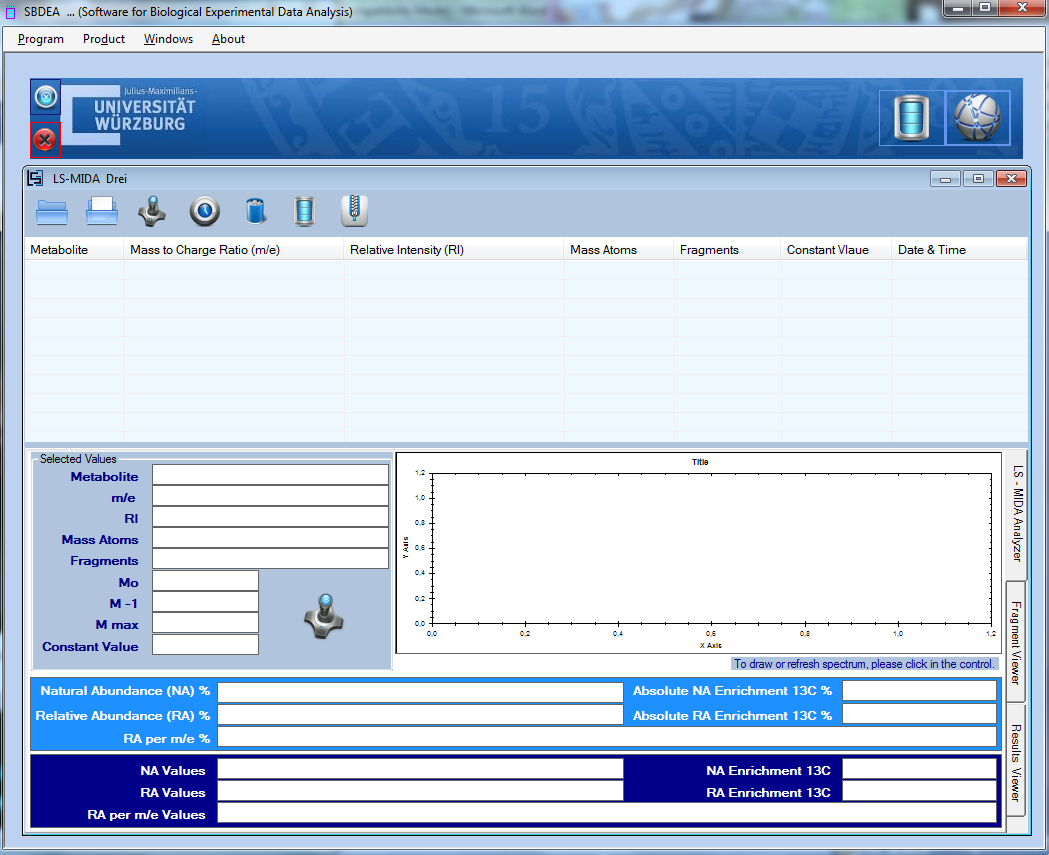


**(B)**


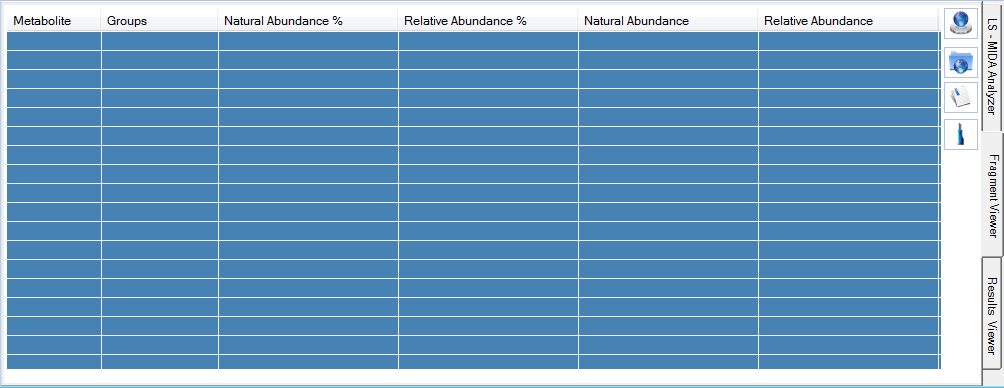


## (C)


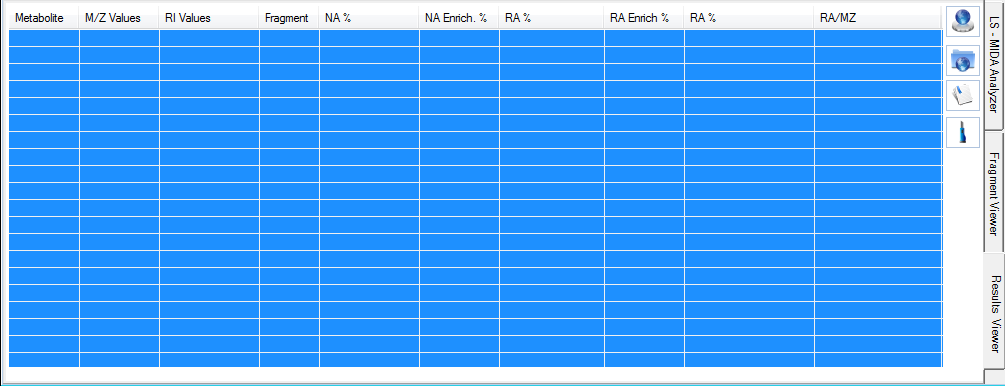


**Figure 1** – **LS-MIDA Data Analyzer Graphical User Interface. (A) Spectrum Analyzer.** **(B) Labelled Isotopomers Viewer.**  **(C) Processed Data Viewer.**


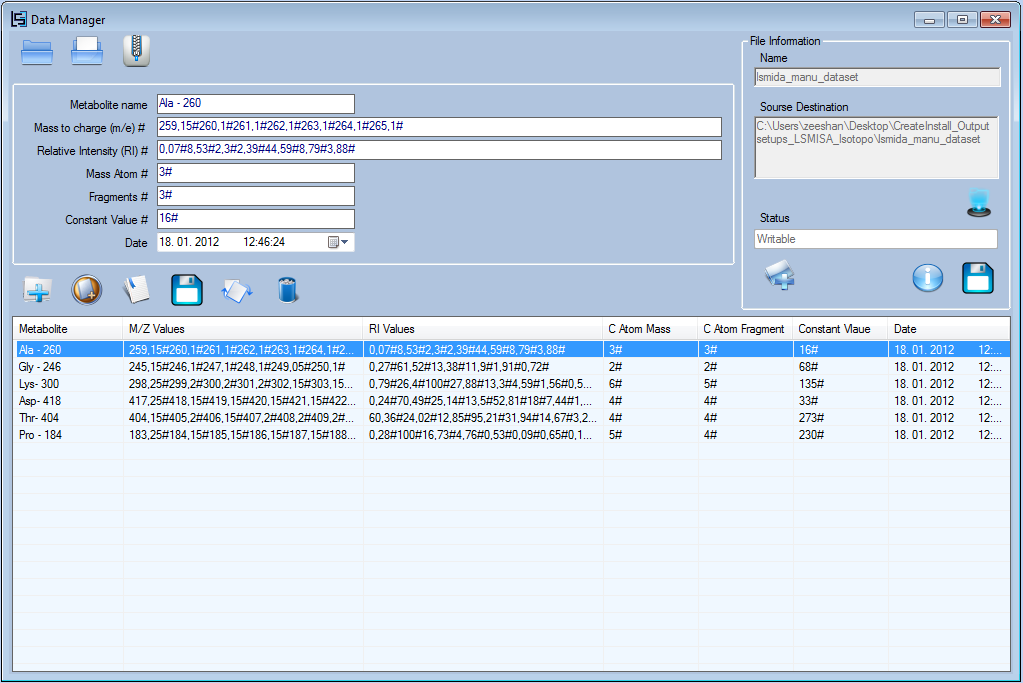


**Figure 2** – **LS-MIDA Data Manager Graphical User Interface.**

**(A)**


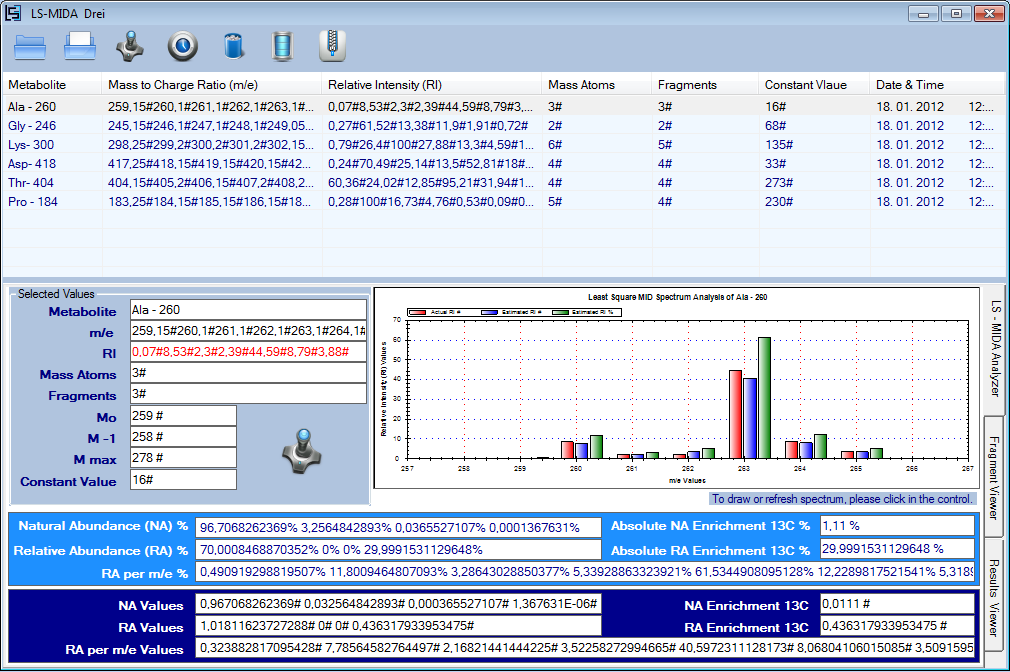


**(B)**


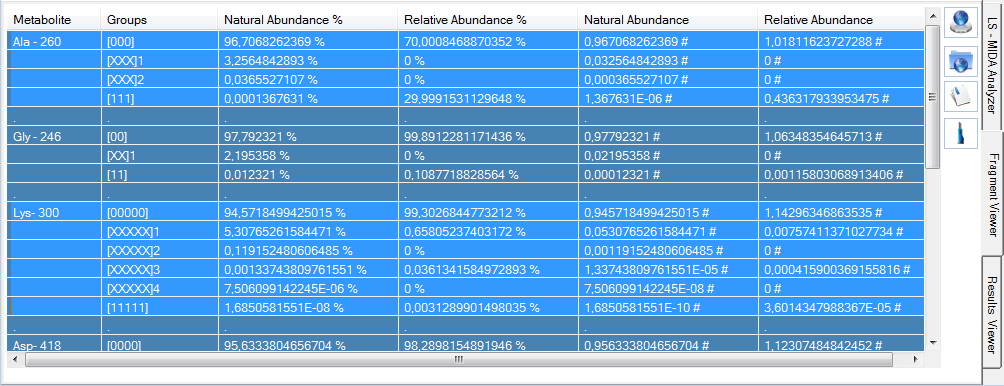


**(C)**


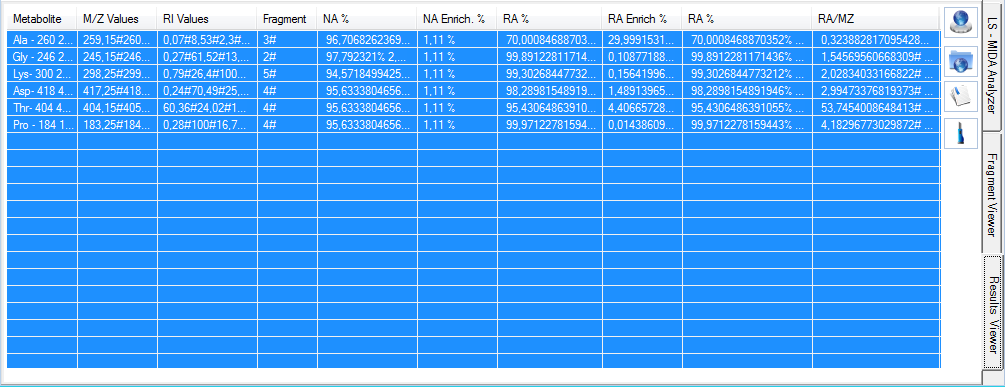


**Figure 3** – **LS-MIDA Data Analyzer (A) Experimental data analysis.** **(B) Estimated Relative Abundance Values**  **(C) Complete input and output information**

**(A)**


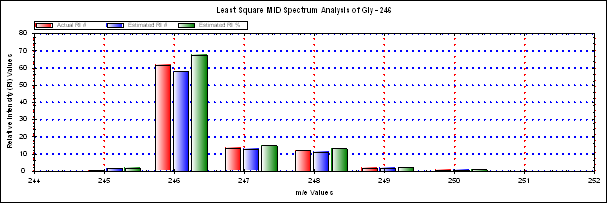


**(B)**


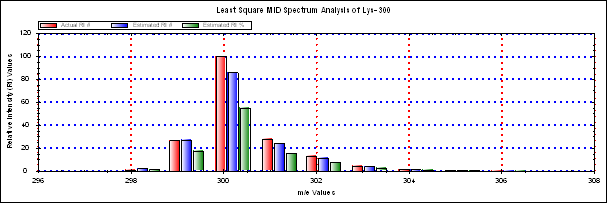


**(C)**


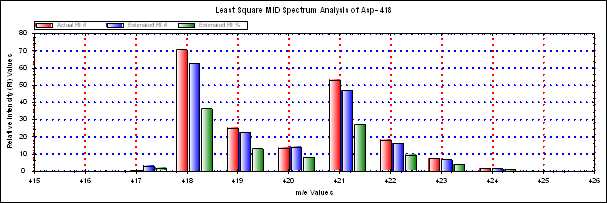


**(D)**


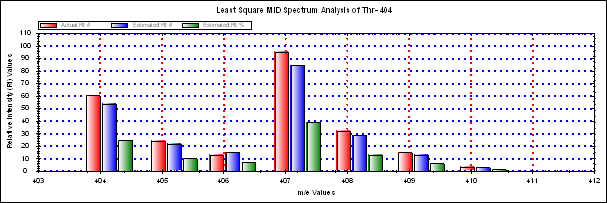


**(E)**


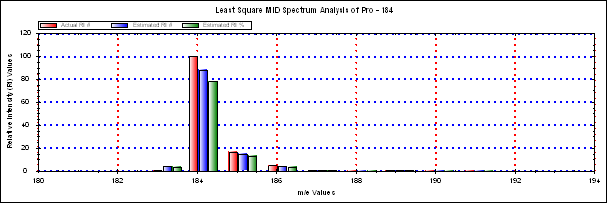


**Figure 4–** **Drawn Spectrums using LS-MIDA Data Analyzer (A) Spectrum Gly 246**

**(B) Spectrum Lys 246. (C) Spectrum Asp 418. (D) Spectrum Thr 404. (E) Spectrum Pro 184.**

**
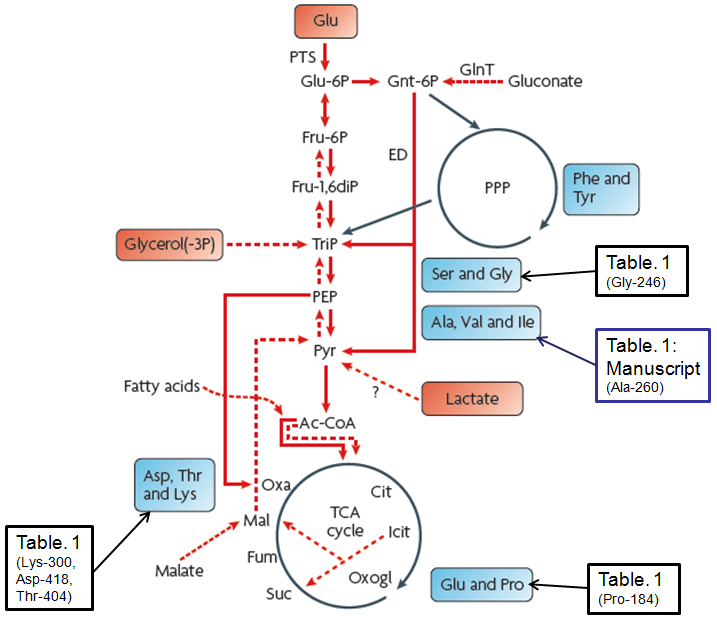
**

**Figure 5–** **Salmonella Model**

Starting from a basic model of Salmonella metabolism [3] we show here current investigations and the position of the data examples given here on the simplified metabolic map of Salmonella.
